# Facial Granulomatous Rosacea: A Case Report

**DOI:** 10.7759/cureus.45391

**Published:** 2023-09-17

**Authors:** Rawan S Almutairi, Humoud Y Al-Sabah

**Affiliations:** 1 Dermatopathology, As'ad K. Al-Hamad Dermatological Center, Kuwait, KWT

**Keywords:** dermatopathology, cutaneous sarcoidosis, lupus miliaris disseminatus faciei, noncaseating granuloma, granulomatous rosacea

## Abstract

Granulomatous rosacea is a chronic inflammatory skin disease. We present the case of a 30-year-old woman with a three-month history of erythematous monomorphic papules and nodules on the cheeks and forehead. Histopathological examinations revealed tuberculoid granulomas with multinucleated giant cells. Granulomatous rosacea should be differentiated from other similar granulomatous skin diseases such as cutaneous sarcoidosis and Lupus miliaris disseminates faciei.

## Introduction

Granulomatous rosacea (GR) is a chronic inflammatory skin disease that predominantly affects women of middle age [1]. This disease presents with persistent erythema and papules that are histopathologically characterized by granulomas and has a delayed therapeutic response relative to other types of rosacea [2]. The pathophysiology is not completely understood, but neurovascular abnormalities and inflammation are suspected. This disease may be triggered by environmental factors such as sun exposure, temperature fluctuations, alcohol consumption, and an inflammatory stimulation by Demodex folliculorum [3]. We present the case of a 30-year-old woman with a three-month history of GR.

## Case presentation

**Figure 1:**
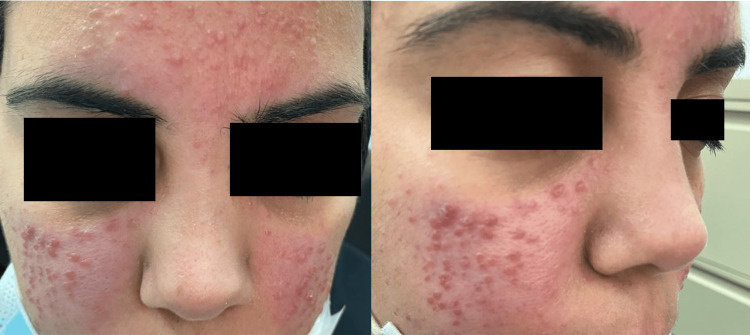
Granulomatous rosacea Multiple monomorphic firm papules with an erythematous background involving both cheeks and the forehead

**Figure 2:**
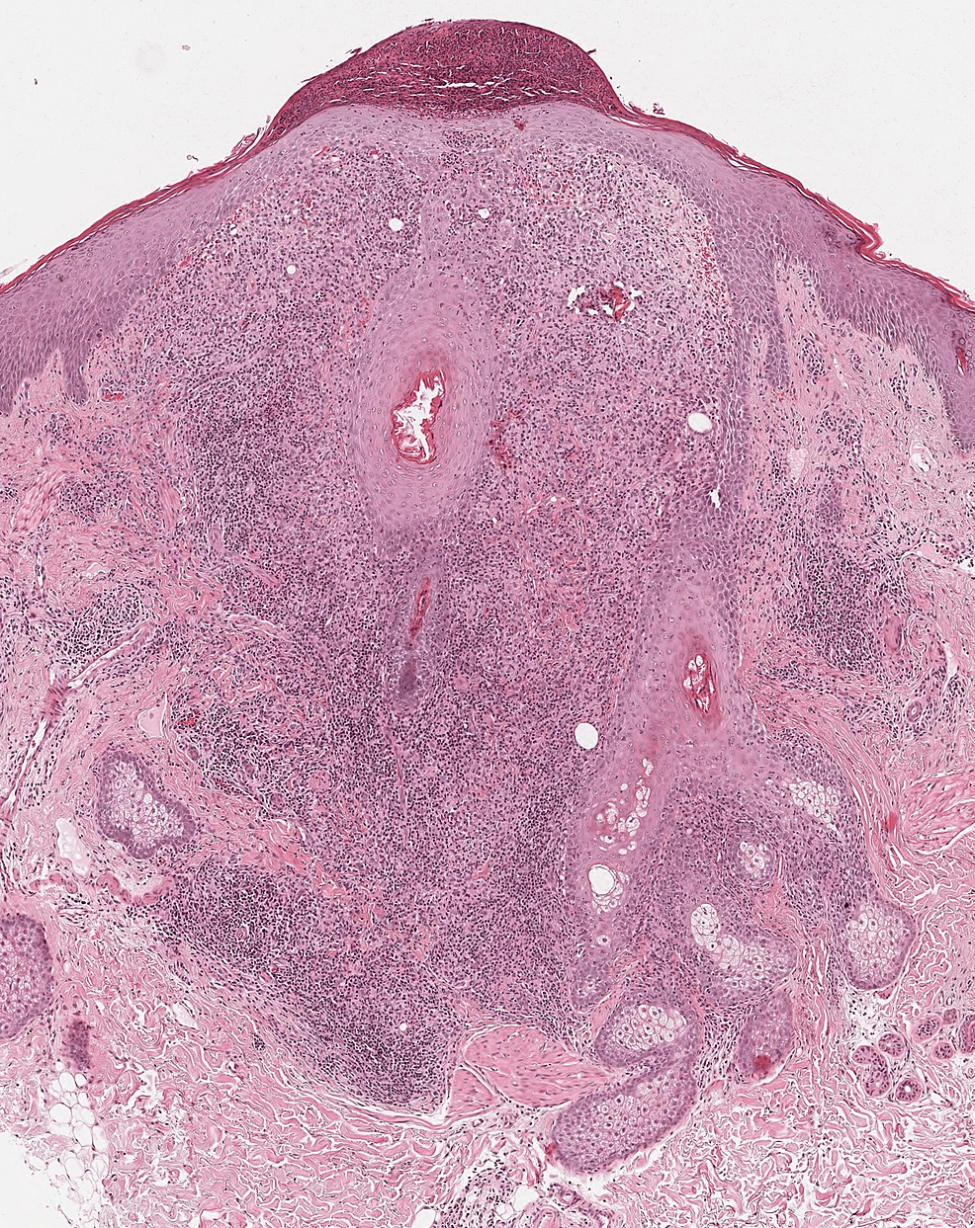
Granulomatous rosacea Histological examination detected perifollicular tuberculoid granulomas composed of lymphocytes, epithelioid histiocytes, and multinucleated giant cells. The superficial blood vessels are dilated with extravasated blood cells and a subcorneal pustule filled with neutrophils. (HE x40)

A 30-year-old healthy female patient presented with a three-month history of erythematous nonpruritic lesions of the face. The patient noted exacerbation with sun exposure and heat. Clinical examination revealed persistent facial erythema with red or flesh-colored monomorphic firm papules and nodules involving both cheeks and the forehead (Figure 1). The patient had no significant personal or family history and had no lesions elsewhere on her body. She reported no systemic symptoms. She was treated with doxycycline with no improvement. Differential diagnoses included GR, cutaneous sarcoidosis, and Lupus miliaris disseminatus faciei (LMDF). Routine laboratory investigations showed normal complete blood counts and renal and liver function tests. A skin biopsy was performed and a histological examination detected perifollicular tuberculoid granulomas composed of lymphocytes, epithelioid histiocytes, and multinucleated giant cells. The superficial blood vessels are dilated with extravasated blood cells and a subcorneal pustule that is filled with neutrophils (Figure 2). Giemsa, Periodic acid-Schiff (PAS), and modified Ziehl-Neelsen (ZN) stains were all negative. The clinical and histological findings were consistent with the diagnosis of GR.

## Discussion

The standardization of rosacea classification in 2002 resulted in the identification of four subtypes and one variant. The four subtypes are papulopustular, phymatous, ocular, and erythematotelangiectatic. GR is recognized as a variant of rosacea [4]. In a more recent classification system, however, GR was omitted [5]. We acknowledge that GR is a subtype of rosacea and not a distinct disease entity.

Clinically, GR can be recognized by the presence of firm, yellow, brown, or red papules or nodules on the skin, which can be severe and cause scarring. They typically appear on the cheeks and periorifices and can vary in size between patients although they are monomorphic within each individual, as observed in our patient. Other rosacea signs are not necessary for diagnosing the GR variant [1,4]. Despite the importance of clinical correlation, the diagnosis of GR is dependent on the histopathologic examination.

Histological examination of the GR can range from a lymphohistiocytic response to the formation of noncaseating epithelioid granulomas. Mullanax and Kierland first described GR in 1970 as a non-caseating epithelioid granuloma with a mixture of epithelioid cells, lymphocytes, and occasional giant cells, which are disease-specific characteristics [6]. In 2004, GR was described histopathologically with three patterns, including small concentrated palisading granulomas, diffuse elastolytic granulomas, and granulomas surrounding follicles and extrafollicular Demodex folliculorum [7]. Demodex folliculorum was identified in seven of 24 GR cases (29%) [8]; its absence does not exclude the disease, as in our case. Our patient manifested centrofacial erythematous papules and nodules, in addition to non-caseating granulomas on histopathology, supporting the diagnosis of GR.

GR must be differentiated from other conditions with comparable clinical and histological characteristics such as cutaneous sarcoidosis and LMDF. Lesions in cutaneous sarcoidosis usually have red-brown to purple maculopapules; however, they can also be skin-colored, yellow-brown, or hypopigmented. In the background of erythema, the papules had a distinctive translucent yellow-brown hue. Diascopy reveals the distinct apple-jelly color of the granulomatous cutaneous lesions. Similar to GR, lesions in cutaneous sarcoidosis can appear anywhere in the body; however, they are most commonly found on the face and neck, particularly on the eyelids, around the orbits, and in the nasolabial folds. The absence of plaques, lupus pernio, subcutaneous infiltrates, and scar infiltration differentiates GR from sarcoidosis [9]. In addition to cutaneous sarcoidosis, LMDF can mimic GR clinically, as it presents with inflammatory, erythematous, flesh-colored papules distributed symmetrically across the eyelids, nose, and upper lip. The papules tended to be numerous, smooth, brownish-red, and 1-3 mm in diameter. Occasionally, the lesions can be extensive and manifest on the extremities and trunk. Erythema surrounding LMDF lesions is not a characteristic feature but may be present [1].

Since diagnosing GR clinically is difficult in the presence of its mimickers, skin biopsy for histopathological examination remains a crucial method in confirming the disease. Histopathologically, LMDF is characterized by dermal epithelioid granuloma with caseous necrosis [10]. On the other hand, in cutaneous sarcoidosis, sarcoidal granulomas are distinct, uniformly distributed in the dermis, and surrounded by a minimal number of lymphocytes, hence named naked granulomas [11].

## Conclusions

We believe that the patient had granulomatous rosacea. With this clinical presentation, all the differential diagnoses mentioned in the discussion must be considered. Histopathological examination remains the gold standard method for distinguishing GR from their mimics.
